# Different culture media affect growth characteristics, surface marker distribution and chondrogenic differentiation of human bone marrow-derived mesenchymal stromal cells

**DOI:** 10.1186/1471-2474-14-223

**Published:** 2013-07-30

**Authors:** Sebastien Hagmann, Babak Moradi, Sebastian Frank, Thomas Dreher, Peer Wolfgang Kämmerer, Wiltrud Richter, Tobias Gotterbarm

**Affiliations:** 1 Department of Orthopedics, Trauma Surgery and Spinal Cord Injury, University Hospital Heidelberg, Germany Schlierbacher Landstrasse 200a, 69118 Heidelberg, Germany; 2 Maxillofacial and Plastic Surgery, University Medical Center, Mainz, Germany; 3 Research Center for Experimental Orthopedics, University Hospital Heidelberg, Heidelberg, Germany

**Keywords:** Mesenchymal stromal cells, Expansion media, Surface markers, Osteogenic differentiation, Chondrogenic differentiation, Adipogenic differentiation

## Abstract

**Background:**

Bone marrow-derived mesenchymal stromal cells (BM-MSCs) play an important role in modern tissue engineering, while distinct variations of culture media compositions and supplements have been reported. Because MSCs are heterogeneous regarding their regenerative potential and their surface markers, these parameters were compared in four widely used culture media compositions.

**Methods:**

MSCs were isolated from bone marrow and expanded in four established cell culture media. MSC yield/1000 MNCs, passage time and growth index were observed. In P4, typical MSC surface markers were analysed by fluorescence cytometry. Additionally, chondrogenic, adipogenic and osteogenic differentiation potential were evaluated.

**Results:**

Growth index and P0 cell yield varied importantly between the media. The different expansion media had a significant influence on the expression of CD10, CD90, CD105, CD140b CD146 and STRO-1. While no significant differences were observed regarding osteogenic and adipogenic differentiation, chondrogenic differentiation was superior in medium A as reflected by GAG/DNA content.

**Conclusions:**

The choice of expansion medium can have a significant influence on growth, differentiation potential and surface marker expression of mesenchymal stromal cells, which is of fundamental importance for tissue engineering procedures.

## Background

Since their recognition in the 1960s and 70s [1,2], mesenchymal stem or stromal cells (MSC) are considered one of the most promising targets for regenerative medicine. MSC were first isolated from animal bone marrow (BM) [3], but a variety of tissues in humans, such as adipose tissue [4], cord blood [5], peripheral blood [6] and connective tissues [7] have also proven to be a yielding source for these cells.

The potency of human MSC (hMSC) to differentiate into various cell lines, such as fibroblasts, myofibroblasts, osteoblasts, chondroblasts and adipocytes [8,9] put these cells into the current focus of tissue engineering, particularly in the fields of bone [10,11] and cartilage [12,13] regeneration, but also in myocardial infarction [14,15].

Minimal criteria for MSC have been postulated by the International Society for Cellular Therapy [16]: they must express CD105, CD73 and CD90 and lack expression of hematopoietic markers such as CD45, CD34, CD14 and CD11b. In addition, MSC must be capable of differentiating into fibroblasts, osteoblasts, adipocytes and chondroblasts under specific *in vitro* conditions.

However, there is an enormous variance in cell isolation techniques and expansion conditions for MSCs [12,17-19]. Basal culture media and supplements have become a considerable market with the growth of mesenchymal stromal cell research. Up to this day, only little attention was paid to the distinct variations of culture media, growth factors and other supplements used in this field of research.

Yet there is increasing evidence that the choice of culture media has an important influence on the biological properties of MSCs [9,19-21]. Besides, recent studies report that bone marrow derived MSC may consist of several distinct subpopulations with diverse regenerative potential [22,23]. However, the properties of these subpopulations and their usability in tissue engineering are only marginally explored.

The aim of this study therefore was to analyse the influence of four different widely used culture media on growth parameters and surface marker distribution of human BM-MSCs. Rather than determining the influence of single culture additives, actual media compositions that were in use in our laboratory were compared to each other with the main focus on the impact on surface marker distribution.

## Methods

### Bone marrow donors

Human MSCs were isolated from bone marrow of 12 donors (mean age 33 ± 21.6 years, 6 female, 6 male). Bone marrow aspiration was performed in the tibia (n = 2), femur (n = 2) or the iliac crest (n = 8) within the framework of total joint arthroplasty or osteotomy. The study protocol had been approved by the ethics committee of the University of Heidelberg, Germany. All patients provided informed consent according to the latest version of the Helsinki Declaration.

### Mesenchymal stromal cells (MSCs)

Bone marrow was intraoperatively diluted to 1:1 isotonic saline solution (B. Braun Melsungen, Germany) with 5000 I.E. of heparine (ratiopharm, Ulm, Germany). Mononuclear cells (MNCs) were isolated from these bone marrow samples by Ficoll Paque plus (GE Healthcare, Uppsala, Sweden) gradient centrifugation. After Ficoll separation, the cells of all donors were distributed into four equal samples and resuspended in different cell culture media as described below at a density of 5 × 10^5^ cells/cm^2^ (equals 2.5 × 10^6^ cells/ml) in T75 cm^2^ cell culture flasks (Nunc, Roskilde, Denmark). MSCs were then cultured under the exact same conditions, at 37°C with 6% CO2 in a humidified thermostat, except for the use of four different culture media:

1. Dulbecco’s modified Eagle’s medium low glucose (DMEM-LG, Invitrogen, Karlsruhe, Germany) with 10% fetal calf serum (FCS, Biochrom, Berlin, Germany) and 1% penicilline/streptomycine (Invitrogen, Karlsruhe, Germany) (referred to as medium A).

2. Alpha minimum essential medium (αMEM) with L-glutamine with 10% fetal calf serum (FCS, Biochrom, Berlin, Germany) and 1% penicilline/streptomycine (Invitrogen, Karlsruhe, Germany) (medium B).

3. A variation of “Verfaillie” medium (medium C) [18], consisting of 547.5 ml Dulbecco’s modified Eagle’s medium high glucose (DMEM-HG, Invitrogen, Karlsruhe, Germany) and L-glutamine (Invitrogen, Karlsruhe, Germany), 40 ml MCDB (Sigma-Aldrich, Steinheim, Germany), 20 ml FCS (Biochrom, Berlin, Germany), 10 ml penicilline/streptomycine (Invitrogen, Karlsruhe, Germany), 20 ml IST (Sigma-Aldrich, Steinheim, Germany), 2 ml dexamethasone (Sigma-Aldrich, Steinheim, Germany), 500 μl ascorbic acid (Sigma-Aldrich, Steinheim, Germany), 10 ng/ml PDGF-BB (Miltenyi Biotec, Bergisch Gladbach, Germany), 10 ng/ml EGF (Miltenyi Biotec, Bergisch Gladbach, Germany).

4. A variation of “Bernese chondrocyte medium” (medium D) [24,25], consisting of Dulbecco’s modified Eagle’s medium F12 + L-glutamine (Invitrogen, Karlsruhe, Germany), 1% penicilline/streptomycine (Invitrogen, Karlsruhe, Germany), 10% FCS (Biochrom, Berlin, Germany), 10 μl/50 ml TGF-ß1 (Acris, Herford, Germany), 2.5 μl/50 ml FGF-2 (Acris, Herford, Germany).

After 24 hours, non-adherent cells were discarded; afterwards, medium replacement was carried out every 72 hours.

Cells were inspected daily for confluence by polarisation microscopy. When reaching 80% confluence, cells were detached by incubation with trypsine (Biochrom, Berlin, Germany), harvested and washed with the corresponding complete medium. Cells were counted in triplicates after staining with trypan blue 0.4% (Sigma-Aldrich, Steinheim, Germany). MSCs were then resuspended and further cultured according to the protocol detailed above. The end of this first passage was defined as P1. The procedure was repeated until the end of the fourth passage (P4).

### Growth parameters

Cells were counted before and after Ficoll separation, and after each passage in triplicates as detailed above, and passage time was analyzed. The following parameters were then calculated: MSC yield in P0, P1, P2, P3 and P4 per 1000 BM-MNCs and MSC growth index (MSCs before passage x/MSCs after passage x) and compared between the media.

### Chondrogenic differentiation

After P4, MSCs were harvested with trypsine/EDTA as described above and centrifuged into pellets containing 5 × 10^5^ cells (n = 6 donors per group, n = 3-5 pellets per donor). Chondrogenic differentiation was induced with a chondrogenic medium consisting of 286 ml DMEM HG (Invitrogen, Karlsruhe, Germany), 150 μl transferrin 10 mg/ml (Sigma-Aldrich, Steinheim, Germany), 1 μl sodium selenite 100 μg/ml (Sigma-Aldrich, Steinheim, Germany), 3 ml Sodium pyruvate 350 mM (Sigma-Aldrich, Steinheim, Germany), 5 ml BSA 7.5% (Invitrogen, Karlsruhe, Germany), 3 ml P/S (Invitrogen, Karlsruhe, Germany), supplemented with 50 μl dexamethasone/5 ml (Sigma-Aldrich, Steinheim, Germany), 5 μl ascorbic acid/5 ml (Sigma-Aldrich, Steinheim, Germany), 5 μl TGF-ß1/5 ml (Acris, Herford, Germany), 6.9 μl insuline glargin/5 ml (Sanofi Aventis, Frankfurt, Germany). Medium was changed three times a week. Chondrogenic differentiation potential was evaluated by a glycosaminoglycan (GAG) assay (n = 2 per donor). The pellets were digested with pepsin solution overnight. 1,9-dimethyl-methylene blue (dye content 80%, Sigma-Aldrich, Steinheim, Germany) was used for staining of glycosaminoglycans. Measurements of absorption were performed at 530 nm and compared to a chondroitin 4-sulfate standard (Sigma-Aldrich, Steinheim, Germany). DNA content was measured with Quant iT ds Pico Green DNA Assay Kit (Invitrogen, Karlsruhe, Germany) according to manufacturers’ protocols.

In addition, the remaining pellets were fixed in 4% paraformaldehyde and cut into 5 μm sections for histology and immunohistochemistry. The sections were either stained with 1% alcian blue (Chroma, Muenster, Germany) and counterstained with fast red (Sigma-Aldrich, Steinheim, Germany), SafraninO/Fast Green (Sigma-Aldrich, Steinheim, Germany) or used for immunohistochemistry. Collagen I and Collagen II staining was performed by pre-treating the sections with 2 mg/ml hyaluronidase (Merck, Darmstadt, Germany) and 1 mg/ml pronase (Roche Diagnostics, Penzberg, Germany). The sections were then subjected to PBS containing 5% BSA in order to block unspecific background and incubated with a monoclonal mouse anti-human collagen type or II antibody (ICN Biomedicals, Aurora, OH) in PBS containing 1% BSA. Reactivity to the antibodies was detected by applying a biotinylated goat anti-mouse secondary antibody (Dianova, Hamburg, Germany), streptavidin-alkaline phosphatase (Dako, Hamburg, Germany), and fast red (Sigma-Aldrich, Steinheim, Germany).

### Osteogenic differentiation

After passage 4, MSCs were harvested with trypsin/EDTA as described above and 35,000 cells per well were seeded in 24 well plates (Nunc, Roskilde, Denmark) containing osteogenic induction medium which consisted of DMEM high glucose (Invitrogen, Karlsruhe, Germany), 10% FCS (Biochrom, Berlin, Germany), 0.1 mM dexamethasone (Sigma-Aldrich, Steinheim, Germany), 0.17 mM ascorbic acid 2-phosphate (Sigma-Aldrich, Steinheim, Germany), 10 mM β-glycerophosphate (Sigma-Aldrich, Steinheim, Germany) and 1% penicilline/streptomycine (Biochrom, Berlin, Germany).

At day 21, osteogenesis was quantified by alkaline phosphatase assay according to standard protocols. Briefly, MSCs were lysed in 0.5 ml 1% Triton X-100 (Sigma-Aldrich, Steinheim, Germany). 100 ml lysate were incubated with 100 ml of 1 mg/ml p-nitrophenylphosphate in ALP-buffer (0.1 M glycine, 1 mM MgCl2, 1 mM ZnCl2, pH 10.4). The substrate turnover was then measured at 405/490 nm using an MRX ELISA reader (Dynatech Laboratories, Stuttgart, Germany). Results were standardized to lysate protein content using a Micro BCA Protein Assay Kit (Pierce, Rockford, USA) according to manufacturers’ instructions.

In addition, calcium deposition was quantified by 0.5% Alizarin Red S staining (Chroma, Münster, Germany) according to standard protocols and quantified at 570 nm. The results were standardized to whole protein content as described above.

### Adipogenic differentiation

After passage 4, MSCs were harvested with trypsin/EDTA as described above and 35,000 cells per well were seeded in 24 well plates (Nunc, Roskilde, Denmark) containing adipogenic induction medium, consisting of DMEM high glucose (Invitrogen, Karlsruhe, Germany), 10% FCS (Biochrom, Berlin, Germany), 1 mM dexamethasone (Sigma-Aldrich, Steinheim, Germany), 0.2 mM indomethacine (Sigma-Aldrich, Steinheim, Germany), 0.5 mM isobutyl methylxanthine (Sigma-Aldrich, Steinheim, Germany), 0.01 mg/ml insulin glargin (Sanofi-Aventis, Frankfurt, Germany) and 1% penicilline/streptomycine (Biochrom, Berlin, Germany).

At day 21 the cells were fixed with 4% paraformaldehyde and stained with 0.3% Oil Red O solution (Chroma, Münster, Germany) to quantify differentiation into adipocyte. Re-extraction of the dye was performed with 60% isopropanol and the optical density was measured at 490 nm.

### Flow cytometry analysis

MSCs were detached with trypsine as described above, washed in whole medium and resuspended in phosphate buffered saline with 0.5% FCS and 2 mM EDTA.

The following anti-human antibodies were used in the experiments: CD105 PE mouse IgG1, CD133 mouse IgG1 (Miltenyi Biotec, Bergisch Glattbach, Germany), CD10 FITC mouse IgG1, CD13 PE mouse IgG1, CD14 FITC mouse IgG1, CD34 PE mouse IgG1, CD44 FITC mouse IgG2b, CD45 FITC mouse IgG1, CD49a PE mouse IgG1, CD90 FITC mouse IgG1, CD140b PE mouse IgG2a CD146 PE mouse IgG1, CD166 PE mouse IgG1, CD271 mouse IgG1 (BD Biosciences, Heidelberg, Germany), CD340 mouse IgG1 (Genway, San Diego, USA), HLA-ABC PE (Dako, Glostrup, Denmark), for MSC analysis. STRO-1 mouse IgM (R + D Systems, Wiesbaden, Germany) was labeled with a secondary goat anti-mouse FITC antibody (Dako, Glostrup, Denmark). Isotype matched control antibodies (IgG1 FITC and PE, IgG2a FITC and PE, IG2b FITC, all Dako, Glostrup, Denmark) were used for assessment of background fluorescence.

One-color and two color cytometry was performed using a FACS Scan® analyser (BD Biosciences, San Jose, USA) and the Cellquest Pro® Software (BD Biosciences, San Jose, USA). Positive fluorescence was defined as any event above the background fluorescence, which was defined by a histogram cut-off where 99.5% of the events in isotype antibody labeled cells were considered negative. All results regarding flow cytometry are displayed as % positive cells.

### Statistical analysis

Statistical analysis was performed using the SPSS computer software (SPSS Inc. Released 2009. PASW Statistics for Windows, Version 18.0. Chicago: SPSS Inc.). A three steps testing of normal distribution was performed for each data set: 1. Graphic display (QQ-plot, histogram and box plot), 2. Ratio analysis, 3. Kolmorogov-Smirnov (with Lilliefors significance correction) and Shapiro-Wilks testing. Analyses of variance (ANOVA) followed by Bonferroni correction were performed to compare the results for parametric data (osteogenic differentiation results). For non-parametric data, Friedman tests were performed to compare the four groups, followed by Wilcoxon tests for the comparison of two groups for paired data (chondrogenic differentiation results). A Kruskal-Wallis test for the comparison of four groups, followed by Mann–Whitney-U-tests was performed for non-paired data (growth parameters, surface markers). Differences were considered statistically significant for p-values smaller 0.05. Results are displayed as means ± standard deviation.

## Results

### MSC growth characteristics are strongly influenced by the choice of expansion media

An important donor-dependency of MSC growth characteristics was observed. However, there was no apparent correlation between growth parameters and the age of the donor or the site of bone marrow harvest.

**Figure 1:**
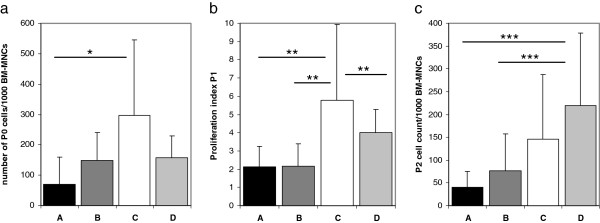
**MSC growth parameters.** Growth parameters of human bone marrow derived MSCs cultured in four different media (see Methods for composition details): medium A (n = 8), B (n = 11), C (n = 11), D (n = 8). **a)** Number of cells after P0 per 1000 BM-MNCs. **b)** Proliferation index in P1 (cells seeded/cells extracted after P1). **c)** P2 MSC yield per 1000 initially seeded BM-MNCs.

**Figure 2:**
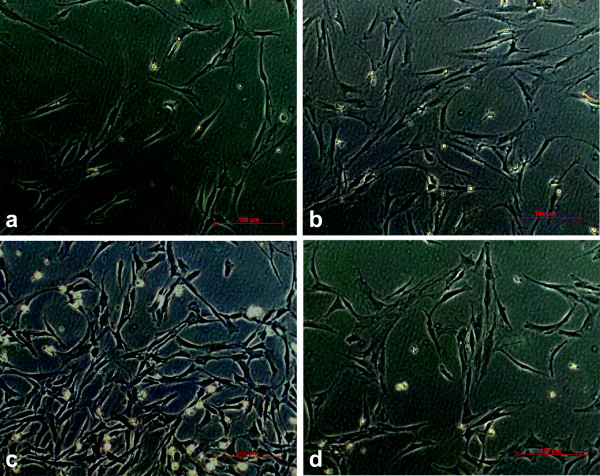
**MSC morphology.** Gross appearance of human BM-MSCs cultured in four different media after 8 days in P0, polarized light microscopy, index 100 μm. **a)** medium A **b)** medium B **c)** medium C **d)** medium D (see Methods for media composition). While all media display a similar cell phenotype typical for plastic adherent MSCs, MSCs cultured in medium C grew faster with earlier confluence.

The amount of MNCs in the bone marrow aspirates ranged from 6.25x10^5^/ml to 1.69x10^7^/ml (mean 3.85 ± 4.9 × 10^6^ cells/ml). Mean MSC yield per 10^3^ BM-MNCs at P0 was significantly higher in medium C when compared to medium A (297.29 ± 248.21 in medium C vs. 70.31 ± 88.09 in medium A; p = 0.018) (Figure 1a). This was also reflected by a higher cell count and earlier confluency in light microscopy when cells cultured in medium C were compared to the other media at the same time points (Figure 2). Four MNC samples in medium A and D and one in medium B and C showed no growth at all.

Cell proliferation rate differed substantially between the media with significantly lower growth index values for medium A, B and D compared to medium C in P1 (Figure 1b, comparison of all groups p < 0.001; 5.77 ± 4.14 for medium C vs. 2.16 ± 1.22 for medium B, 2.2 ± 1.02 for medium A, 3.9 ± 1.27 for medium C; p = 0.0033, p = 0.0027 and p = 0.0087, respectively). In P2, no significant differences between the media were observed regarding the growth index (comparison of all groups p = 0.32) due to the fact that proliferation in medium C was significantly reduced in P2 when compared to P1 (2.35 ± 1.57 in P2 vs. 5.77 ± 4.14 in P1, p = 0.014). Interestingly, this resulted in medium D showing the highest cell yield per 1000 BM-MNCs after P2 (Figure 1c, 219.37 ± 158.32 for medium D vs. 41.14 ± 33.9 for medium A, p < 0.001; vs. 76.54 ± 81.52 for medium B, p = 0.005; comparison of all groups p = 0.01). No significant differences in proliferation were observed between any of the culture conditions tested after P2.

Passage time in P0 was significantly lower in medium C and medium B compared to medium A (comparison of all groups p = 0.021; 12 ± 2.13 days in medium C and 12.75 ± 2.34 days in medium B vs. 17.58 ± 5.98 in medium A, p = 0.004 and p = 0.012, respectively). Although medium C showed the lowest mean passage time in P1, this trend did not reach significance (data not shown, p = 0.096). In P2, medium C showed the fastest passage time with a significant difference to medium B (3.91 ± 1.3 days vs. 7.25 ± 2.63 days, p < 0.001; comparison of all groups p = 0.003). Regarding passage time, no significant differences were observed after P2.

### Distribution of several BM-MSCs surface markers is dependent on expansion media

**Figure 3:**
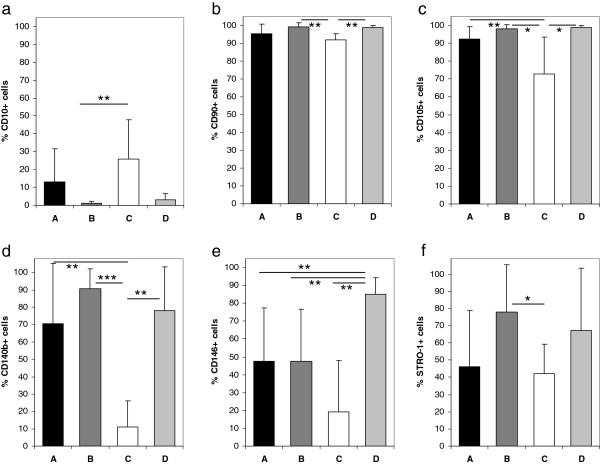
**MSC surface marker distribution.** Differences in surface marker distribution between of human BM-MSCs cultured in four different media (see Table 1 for data). All results are displayed as % positive cells, means of n = 10 for medium B and C and n = 6 for medium A and D. **a)** CD10, **b)** CD90, **c)** CD105, **d)** CD140b, **e)** CD146, **f)** STRO-1.

Fluorescence cytometry of MSC surface markers allowed distinction into clearly “positive”, clearly “negative” and intermediate MSC markers (Table 1, Figure 3). In all media, there was a strong fluorescence signal (“positive” markers) for CD13, CD44, CD73, CD90, CD105 and CD166 (Table 1, Figure 3). There was an important variation of CD105 fluorescence in medium C, resulting in a significantly lower mean fluorescence compared to the other media (comparison of all groups p = 0.0011; 72.69 ± 20.72% in medium C vs. 98.29 ± 2.04% in medium B, p = 0.012 and 98.79 ± 1.22% in medium D, p = 0.015). A significantly reduced fluorescence for CD90 was also found in medium C when compared to medium B and medium D (comparison of all groups p = 0.001, 91.89 ± 3.65% for medium C vs. 99.12 ± 2.24% for medium B, p = 0.003 and 98.77 ± 1.39% for medium D; p = 0.002, respectively). CD166 fluorescence was reduced in medium C when compared to medium D (p = 0.026). There was no difference between the media regarding CD13, CD44 and CD73 fluorescence.

**Table 1:** Surface marker distribution on human bone marrow derived mesenchymal stromal cells in four different culture conditions

	**Medium A**	**Medium B**	**Medium C**	**Medium D**
**CD10**	12.954 ± 18.76	1.02 ± 1.35	26 ± 21.84	3.15 ± 3.44
**CD13**	95.37 ± 8.08	98.97 ± 2.03	99.83 ± 0.11	99.79 ± 0.27
**CD14**	0.36 ± 0.19	1.21 ± 1.28	0.47 ± 0.22	0.63 ± 0.2
**CD34**	0.40 ± 0.42	0.31 ± 0.25	0.32 ± 0.13	0.34 ± 0.15
**CD44**	94.04 ± 8	98.27 ± 1.99	96.13 ± 4.47	99.22 ± 0.6
**CD45**	0.342 ± 0.22	0.33 ± 0.18	0.36 ± 0.17	0.2 ± 0.16
**CD49**	52.03 ± 22.36	61.37 ± 18.11	45.15 ± 31.27	48.23 ± 23.98
**CD73**	95.572 ± 4.19	98.1 ± 1.95	97.57 ± 2.71	99.58 ± 0.26
**CD90**	95.25 ± 5.68	99.12 ± 2.24	91.89 ± 3.65	98.77 ± 1.39
**CD105**	92.32 ± 6.98	98.29 ± 2.04	72.69 ± 20.72	98.79 ± 1.22
**CD133**	0.57 ± 0.32	0.71 ± 1.1	0.74 ± 0.15	0.68 ± 0.61
**CD140b**	70.42 ± 34.91	90.51 ± 11.44	10.87 ± 15	78.12 ± 25
**CD146**	47.64 ± 29.68	47.64 ± 28.96	19.27 ± 28.6	84.92 ± 9.51
**CD166**	90.49 ± 12.19	93.21 ± 9.34	90.16 ± 8.11	99.25 ± 0.81
**CD271**	1.08 ± 1.03	1.8 ± 2.71	2.68 ± 2.34	2.69 ± 2.6
**CD340**	44.4 ± 36.34	69.96 ± 32.97	39.31 ± 24.86	68.61 ± 35.31
**STRO-1**	48.26 ± 32.42	78.11 ± 27.47	42.07 ± 17.09	67.07 ± 36.24
**HLA-ABC**	92.02 ± 17.18	97.13 ± 5.38	90.62 ± 10.83	99.76 ± 0.17

Results are displayed as means of % positive cells ± standard deviation.

Low fluorescence (“negative” markers) was observed for CD10, CD14, CD34, CD45, CD133 and CD271 (Table 1). No significant differences between the media were observed except for CD10 fluorescence in medium C compared to medium B (Figure 3, 26 ± 21.84% vs. 1.02 ± 1.35%, p = 0.01).

Numerous surface markers were found to be highly donor dependent (CD49, CD140b, CD146, CD340 and STRO-1), however, despite this fact, significant differences between the media were observed.

CD146 showed intermediate fluorescence in both medium A and medium B with about 50% of the cells being positive for this marker. Of all media, medium C showed the lowest expression of CD146 while expression was significantly higher in medium D (comparison of all groups p = 0.005; 84.92 ± 9.51% in medium D vs. 19.27 ± 28.6% in medium C, p = 0.001; 47.64 ± 29.68 in medium A, p = 0.003; 47.64 ± 29.96 in medium B, p = 0.016; Figure 3). CD140b expression was also found to be significantly lower in medium C when compared to the other media (comparison of all groups p = 0.003; 10.87 ± 15% vs. 70.42 ± 35% in medium A, p = 0.007; 90.51 ± 11.44% in medium B, p < 0.001; 78.12 ± 25% in medium D, p = 0.004; Figure 3). No significant differences were observed for CD49 and CD340 (Table 1). There was also a lower STRO-1 expression in medium C when compared to medium B (comparison of all groups p = 0.047; 78.11 ± 27.47% in medium B vs. 42.07 ± 17.09% in medium C, p = 0.022; Figure 3).

### The choice of expansion media affects MSC chondrogenic differentiation potential

**Figure 4:**
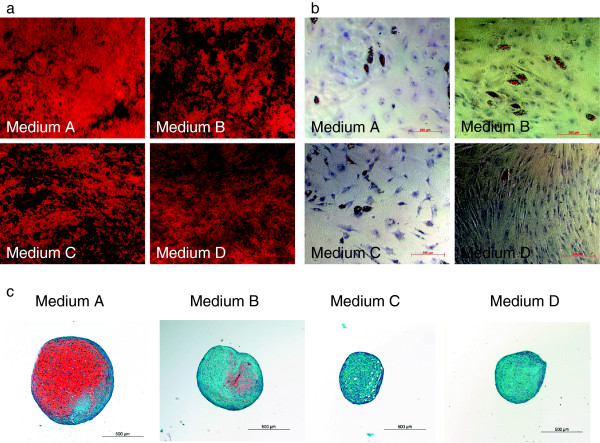
**MSC differentiation.** Influence of four expansion media on osteogenic, adipogenic and chondrogenic differentiation. Light microscopy. **a)** Alizarin Red staining, index 200 μm. **b)** Oil Red O staining, index 200 μm. **c)** Safrananin O/Fast Green staining, index 500 μm. MSCs from six donors were successfully differentiated into chondrogenic, adipogenic and osteogenic lineage. Representative results are shown.

**Figure 5:**
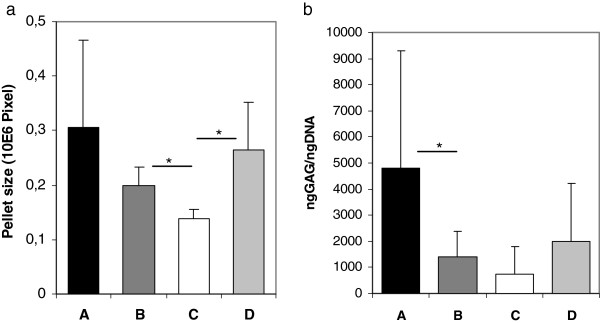
**MSC chondrogenic differentiation.** Chondrogenic differentiation results of human BM-MSCs cultured in different media. **a)** Pellet size after expansion in four different media, n = 6 donors per group, experiments conducted in duplicates. **b)** GAG/DNA content in pellets after expansion in four different media, n = 6 donors per group, experiments conducted in duplicates.

In all media, MSCs were able to differentiate into chondrogenic, osteogenic and adipogenic lineages (Figures 4 and 5). No significant differences between the media were observed regarding osteogenic differentiation (Table 2; Alizarin per well p = 0.455, ALP per protein p = 0.48). Pellet size was significantly lower in medium C (0.14 ± 0.017 × 10^6^ pixel) when compared to medium B (Figure 5a, 0.2 ± 0.033 × 10^6^ pixel, p = 0.028) and medium D (0.27 ± 0.086 × 10^6^ pixel, p = 0.046) and highest in medium A, yet there was no significant difference between this medium and the other media. Chondrogenic differentiation as reflected by GAG/DNA content in the pellets was highest in medium A compared to the other media and lowest in medium C, the differences however were only significant between medium A and B (4786.11 ± 4523.34 in medium A vs. 1424.87 ± 975.83 in medium B, p = 0.046; Figure 5). This difference in chondrogenic differentiation between the media was also reflected by histology (Figure 4c), although Safranin O/Fast Green staining revealed donor-dependent variations in all groups.

**Table 2:** Quantitative results of osteogenic differentiation of human bone marrow derived mesenchymal stromal cells expanded in four different culture conditions

	**Medium A**	**Medium B**	**Medium C**	**Medium D**
**μg Alizarin/well**	174.96 ± 67.47	235.6 ± 98.79	242.48 ± 145.24	285.19 ± 147.29
**ALP per protein**	1.29 ± 1.38	1.42 ± 0.95	0.26 ± 0.42	0.76 ± 0.48

Results are displayed as means ± standard deviation.

## Discussion

After less than two decades of basic research on their proliferation and differentiation potential as well as their immunosuppressive properties, MSCs have entered clinical applications in regenerative medicine, with numerous clinical trials in the fields of orthopaedics, hematology, neurology, cardiology, dentistry and in the treatment of genetical disorders [26-31]. However, there are important variations of isolation and expansion techniques throughout each laboratory, and reports of MSC subtypes with distinct properties are increasing [22,23]. In this context, our study investigated the effect of different currently used expansion media on proliferation, differentiation, and most importantly on surface marker distribution of human bone marrow derived MSCs.

Our data clearly demonstrate that the choice of expansion media can significantly alter not only BM-MSC growth characteristics, but also their differentiation potential such as the ability to form cartilaginous tissue in vitro. Our data are in line with findings of previous studies that have demonstrated inferior chondrogenic differentiation results of MSCs expanded in a control medium (“Verfaillie”, which corresponds to medium C in our study) when compared to ES medium [21]. While ALP per protein was lower in medium C than the other media, no significant differences could be observed between the media, which on the one hand can be explained by the high variation among the donors, and on the other hand by a generally poor osteogenic differentiation in our population. The same could be observed for adipogenic differentiation, which was generally poor, while variations among the donors could be observed. The reasons for these poor differentiation results may lie within the donors or the media preparations. However, all cell preparations were treated in the exact same way, thus indicating that in this context, chondrogenic differentiation potential is more affected by the choice of expansion media than adipogenic and osteogenic differentiation.

Our study adds to findings that quantify differences in surface marker expression depending on the expansion media applied [32]. An important study in this field was published in 2006, revealing the impact not only of the choice of expansion media and growth factors, but also of basic factors such as plating density and flask manufacturer on MSC characteristics [19]. The experiments conducted in this study also revealed that certain MSC surface markers (CD44, MAB1470, STRO-1 and HLA-DR) showed intermediate fluorescence when MSCs were expanded in DMEM-HG, however without quantifying these results. Our study reveals characteristic changes in the distribution of CD10, CD140b and CD146 that to our best knowledge have not been published yet, with consistent results of some more stable markers such as CD14, CD34 and CD45 as “negative markers” and CD13, CD44, CD73 and CD166 as “positive” markers [33].

Another recent study has compared four different expansion media (among these DMEM and Alpha-MEM) and analysed adipose-tissue derived MSCs regarding their surface markers, concluding that the expression of CD49d, CD54 (being lowest in DMEM-KO) and CD117 was inconsistent at all passages and in all four media while the other surface markers did not significantly alter between the media [34]. This is not in accordance with other studies [32] and our own findings that clearly indicate significant differences in the expression of CD10 (not tested in the above study), CD90, CD105, CD140b (not tested in the above study) and CD146 (not tested in the above study). This discrepancy could be either due to differences in the study population or by assuming different characteristics of adipose-tissue derived stem cells [35-37], as well as by the lower number of donors (n = 3) used in the above study. Differences may also be due to the fact that dot charts were employed to analyse fluorescence without indication of a quantitative threshold. In our study, histograms and a threshold of 99.5% were employed to determine positive fluorescence, thus allowing to unmask subtle differences in fluorescence between the media. Another reason for the discrepancy between both studies may be the use of growth factors in two of our media, revealing an important notion of how expansion media may pre-determine the selection of distinct subtypes of MSC through the use of growth factors.

Growth factors such as FGF-2 (or bFGF) and TGF-ß have a long tradition in tissue engineering for modifying the differentiation potential of MSCs. For instance, expansion in the presence of FGF-2 is known to increase proliferation and chondrogenic potential of MSCs [38-40], but to inhibit chondrogenesis when applied during chondrogenic differentiation [41]. Application of FGF-2 to human BM-MSCs during expansion was also found to enhance osteogenesis and adipogenesis while suppressing neuronal differentiation [19]. TGF-ß isoforms have been extensively investigated for their potential to enhance chondrogenesis and osteogenesis [42-44].

However, in our experiments, the highest chondrogenic potential was obtained in a non-growth factor enhanced medium (medium A). This controversy may be due to the fact that differentiation was induced after P4. It is known that the influence of growth factors diminishes with higher passages [38]. Also, the effect cannot be extrapolated on presence or absence of growth factors, as all tested media contained fetal calf serum, which shows such a high variability that most groups select specifically pre-screened lots. In our experiments, the same FCS lot was used for expansion in all four culture conditions. Three of the four media contained 10% FCS; however, growth indices and P0 cell yield/1000 BM-MNCs were highest in the variation of medium C containing 2% FCS, which may be due to its growth factor content. Although the growth factor content may also vary with different FCS lots, in this particular setting we thus directly could demonstrate that application of growth factors does not guarantee control of differentiation.

The use of these easy-to-handle bovine components also remains controversial due to their potential safety risks. Although several reports have propagated easy and efficient use of serum-free or xeno-free MSC expansion media [17,45,46], none of these media have made it to a wide distribution, somewhat reflecting our own experience that the use of these media is still not as practicable as advertised.

The importance of our results lies within the notion that the quality of MSC application may be deeply altered by factors such as the choice of expansion media and the application of growth factors. Moreover, despite ongoing attempts of characterising MSCs and their subtypes, these cells still represent some sort of “black box” hiding properties that are not yet fully understood. It would seem that this knowledge should urge scientist to produce more reproducible results. However, apart from a few studies, no general attempts have been made to standardize MSC isolation and expansion procedures, although it is increasingly understood that MSC may exert different characteristics depending on which laboratory they are cultured in [47]. The minimal MSC-defining criteria made by the International Society for Cellular Therapy [16] have served to narrow the term “mesenchymal stem cell”, however, they lack clarification of a) what is considered presence or absence of typical surface markers and b) how differentiation results can be compared to each other in a quantitative way, considering that numerous reports have outlined that differentiation properties may vary with distinct subtypes of MSCs, expansion and differentiation protocols and different tissues MSCs are derived from.

### Limitations

Our experiments were characterized by an important variation of most parameters dependent on the donor of MSCs. A limitation of this study may be that the age of the donors was widely scattered; however, the influence of age and comorbidities on MSC expansion and differentiation is most controversially discussed [48]. In a small series of patients, our own findings suggested that the choice of culture media was more important than age and comorbidity [49]. Recent reports with larger numbers of patients reveal that proliferation characteristics may decrease with age, but that their lineage choice remains unaltered [48]. Although age may have influenced our findings, by distributing MSCs of all patients and culturing them in all four media, we were able to detect differences that could be solely assigned to the choice of culture media. However, we were able to observe an important variation among the donors that could not be attributed to age. The heterogeneity of MSC preparations of different donors is a known problem [50] that may have had an important influence on growth parameters and differentiation, but surface markers as well.

It could be criticized that two media containing different growth factors were examined along with two media without growth factors and that three media contained 10% FCS while one contained 2% FCS. However, in this study, our aim was to compare four media that are actually widely used in different laboratories rather than to determine the influence of each component. In our opinion, this approach creates a clearer image of how allegedly trivial decisions may have an influence on the outcome of an experiment. It is clear that in the future, experiments attributing the observed differences to single media components will have to be conducted.

## Conclusions

This study adds to several reports that emphasize on how the choice of expansion media can influence not only growth characteristics, but also differentiation potential of MSCs. This study is in accordance with previous findings reporting differences in the expression of typical MSC markers depending on the media applied. We were able to detect additional MSC markers affected by the expansion conditions that to our best knowledge have not been reported yet. Our findings suggest that attempts to further investigate how different media components affect MSC characteristics are necessary. More importantly, our findings are in favour of pursuing standardized protocols in MSC isolation and expansion.

## Abbreviations

Alpha-MEM: Alpha minimum essential medium; BM: Bone marrow; CD: Cluster of differentiation; DMEM-LG: Dulbecco’s modified Eagle’s medium low glucose; FCS: Fetal calf serum; FGF-2: Fibroblast growth factor 2; GAG: Glycosaminoglycans; hMSCs: Human mesenchymal stromal cells; MNCs: Mononuclear cells.

## Competing interests

The authors declare that they have no competing interests.

## Authors’ contributions

SH, BM, WR and TG conceived of the study. SH drafted the manuscript. SH, TD, TG, BM and SF provided the bone marrow. SH and SF carried out the experiments. SF, WR, PWK and SH performed the statistical analysis. SH, WR, BM, TD, PWK and TG participated in study design and coordination and helped to draft the manuscript. All authors read and approved the final manuscript.

## Pre-publication history

The pre-publication history for this paper can be accessed here:

http://www.biomedcentral.com/1471-2474/14/223/prepub
